# Aqueous iron-based redox flow batteries for large-scale energy storage

**DOI:** 10.1093/nsr/nwaf218

**Published:** 2025-05-31

**Authors:** Cailing He, Yiming Zhang, Shuangbin Zhang, Xiyue Peng, Jens Noack, Maria Skyllas-Kazacos, Lianzhou Wang, Bin Luo

**Affiliations:** Australian Institute for Bioengineering and Nanotechnology, The University of Queensland, Brisbane, QLD 4072, Australia; Australian Institute for Bioengineering and Nanotechnology, The University of Queensland, Brisbane, QLD 4072, Australia; Australian Institute for Bioengineering and Nanotechnology, The University of Queensland, Brisbane, QLD 4072, Australia; Australian Institute for Bioengineering and Nanotechnology, The University of Queensland, Brisbane, QLD 4072, Australia; Australian Institute for Bioengineering and Nanotechnology, The University of Queensland, Brisbane, QLD 4072, Australia; German-Australian Alliance for Electrochemical Technologies for Storage of Renewable Energy, The University of New South Wales, Sydney, NSW 2052, Australia; Department of Applied Electrochemistry, Fraunhofer Institute for Chemical Technology, Pfinztal 76327, Germany; School of Chemical Engineering, The University of New South Wales, Sydney, NSW 2052, Australia; Australian Institute for Bioengineering and Nanotechnology, The University of Queensland, Brisbane, QLD 4072, Australia; School of Chemical Engineering, The University of Queensland, Brisbane, QLD 4072, Australia; Australian Institute for Bioengineering and Nanotechnology, The University of Queensland, Brisbane, QLD 4072, Australia

**Keywords:** iron, flow batteries, dissolution-deposition, all-soluble, energy storage

## Abstract

The rapid advancement of flow batteries offers a promising pathway to addressing global energy and environmental challenges. Among them, iron-based aqueous redox flow batteries (ARFBs) are a compelling choice for future energy storage systems due to their excellent safety, cost-effectiveness and scalability. However, the advancement of various types of iron-based ARFBs is hindered by several critical challenges, including hydrogen evolution, inferior reversibility of metal deposition and stripping, and undesirable dendrite formation in hybrid flow systems with metal plating/stripping on the negative electrode. Additionally, all-soluble iron-based ARFBs face limitations in redox species solubility and electrolyte stability. To address these issues, various strategies have been developed, such as modifications to electrolytes, electrodes and separators, as well as flow stack optimization. This review provides a comprehensive overview of iron-based ARFBs, categorizing them into dissolution-deposition and all-soluble flow battery systems. It highlights recent advancements in the field and explores future prospects, focusing on four key areas: materials innovation and mechanistic understanding; flow battery system design and engineering; new electrochemistry explorations; and interdisciplinary strategies. By offering insights into these emerging directions, this review aims to support the continued research and development of iron-based flow batteries for large-scale energy storage applications.

## Teaser

Iron-based aqueous redox flow batteries are emerging as a promising, low-cost option for large-scale energy storagethis review explores recent progress and future opportunities in making them more practical and efficient.

## INTRODUCTION

The continuous growth in global population, rapid lifestyle changes and dramatic economic expansion have significantly accelerated energy demand [1]. While fossil fuels remain a primary energy source, they are insufficient to meet daily energy requirements of many developing countries and pose severe environmental challenges, promoting urgent global action. These concerns have driven the development of renewable energy sources, including tidal, geothermal, solar, wind and biomass energy [2]. Among them, wind and solar energy, as intermittent energy sources, are heavily influenced by time and weather conditions. To address the inherent volatility of renewable energy, the development of reliable electricity energy storage systems is essential [3]. Cost-effective aqueous redox flow batteries (ARFBs) have emerged as a promising option for long-term grid-scale energy storage, enabling stable energy storage and release. The low viscosity of aqueous solutions and high solubility of most chemicals in water strengthen the ionic conductivity of the electrolyte and reduce the cost associated with electrolyte pumping [3]. In addition, the continuous flow of electrolyte in ARFBs effectively alleviates irregular temperature fluctuations and decreases battery polarization compared with conventional static batteries [3]. Importantly, emergency pump deactivation further enhances the safety of this energy storage application.

In 1949, the initial concept of the redox flow battery (RFB) was patented by Kangro and further refined during the development of iron–chromium (Fe–Cr) RFBs by Thaller's group in the National Aeronautics and Space Administration (NASA) [4]. Since then, flow batteries have ushered in an era of rapid development and have become one of the most popular energy storage technologies in recent years. All-vanadium (V) flow batteries (VFBs) have dominated commercialization and industrialization, with several GWh of VFBs already installed in China and elsewhere [5]. However, the instability in the supply and price of vanadium metal has limited its more widespread uptake to date. While new vanadium resources are being developed for near-term commercial production, researchers around the world are exploring other flow-battery chemistries that use more widely available raw materials. Therefore, significant efforts have been directed toward exploring alternative ARFBs based on low-cost materials, such as zinc, iron and organic compounds. Among these, iron-based ARFBs have garnered particular attention in recent years due to the high abundance of iron, its inherent safety, environmental friendliness and cost-effectiveness. Although non-aqueous iron-based flow batteries offer a larger electrochemical operating window, the difficult issues of low operating current density, electrolyte crossover, limited solubility and poor solvent conductivity etc. have not been effectively addressed. Thus, the cost-effective aqueous iron-based flow batteries hold the greatest potential for large-scale energy storage application.

**Figure 1.**
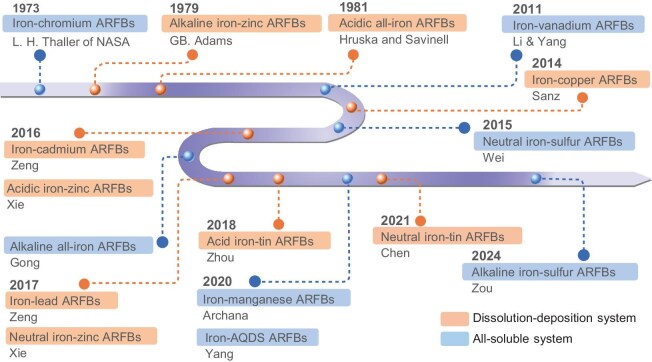
The timeline of birth of 16 types of iron-based ARFBs.

Figure 1 summarizes the development timeline of 16 types of iron-based ARFBs, which are categorized into dissolution-deposition (D-D) iron-based ARFBs and all-soluble (A-S) iron-based ARFBs based on the valency changes of redox pairs during charging and discharging (Table 1). While existing review articles usually focus on one or more core components of the flow battery, or provide a general overview of a few types of iron-based ARFBs [3,5–8], this review offers a comprehensive examination of the key challenges and latest research progress of all iron-based ARFBs to date. Furthermore, it highlights the breakthroughs of acidic and alkaline/neutral all-iron ARFBs, wide pH range iron–zinc (Fe–Zn) ARFBs, iron–tin (Fe–Sn) ARFBs and Fe–Cr ARFBs, with a focus on modifications to three core components of ARFBs: electrolyte, electrode and membrane. These advancements demonstrate the potential of AFRBs for large-scale energy storage applications. Ultimately, advanced modification strategies for future research routes are proposed in terms of the four key areas: materials innovation and mechanistic understanding, flow battery system design and engineering, new electrochemistry explorations, as well as interdisciplinary strategies. This review aims to provide valuable insights into the development of reliable, stable, and high-efficiency iron-based ARFBs for future energy storage systems.

**Table 1.** The respective redox reactions of iron-based ARFBs.

Types of ARFBs (D-D, A-S)		Overall cell reaction	Overall cell voltage (V)	Ref
D-D	Acidic all–iron	* $\rm 3F{e}^{2 + } \rightleftharpoons \rm F{e}^0 + \ \rm 2F{\rm e}^{3 + }$ *	1.21	[9]
D-D	Acidic iron–zinc	$\rm 2F{e}^{2 + } + \ \rm Z{n}^{2 + } \rightleftharpoons \rm Z{n}^0 +\rm 2F{e}^{3 + }$	1.53	[10]
D-D	Alkaline iron–zinc	$$\begin{array}{@{}l@{}} 2{[ \rm {Fe{{( \rm {CN} )}}_6} ]}^{4 - } + \rm Zn{( \rm {OH} )}_4^{2 - } \rightleftharpoons \\ \rm Z{n}^0 + \ \rm 4O{\rm H}^ - + 2{[ \rm {Fe{{( \rm {CN} )}}_6} ]}^{3 - } \end{array}$$	1.58	[11]
D-D	Neutral iron–zinc	$$\begin{array}{*{20} {l}} {2{\rm F{e}}^{2 + } + \ {{\rm Z{n}}}^{2 + } \rightleftharpoons {{\rm Z{n}}}^0 + \ {2{\rm F{e}}}^{3 + }} \end{array}$$	1.43	[12]
D-D	Acidic iron–tin	$\rm {2F{e}}^{2 + } + \ \rm {S{n}}^{2 + } \rightleftharpoons \rm {S{n}}^0 + \ \rm {2F{e}}^{3 + }$	0.90	[13]
D-D	Neutral iron–tin	$$\begin{array}{@{}l@{}} 2{[ \rm {Fe{{( \rm {CN} )}}_6} ]}^{4 - } + \rm S{n}^{2 + } \rightleftharpoons \\ \rm S{n}^0 + 2{[\rm {Fe{{( \rm {CN} )}}_6} ]}^{3 - } \end{array}$$	0.75	[14]
D-D	Iron–lead	$\rm 2F{e}^{2 + } + \rm P{b}^{2 + } \rightleftharpoons \rm P{b}^0 + \rm 2F{e}^{3 + }$	0.90	[15]
D-D	Iron–cadmium	$\rm 2F{e}^{2 + } + \rm C{d}^{2 + } \rightleftharpoons \rm C{d}^0 + \rm 2F{e}^{3 + }$	1.17	[16]
D-D	Iron–copper	$\rm 2F{e}^{2 + } + \rm C{u}^{2+} \rightleftharpoons \rm C{u}^0 + \rm 2F{e}^{3 + }$	0.25	[17]
A-S	Alkaline all-iron	$$\begin{array}{@{}*{1}{l}@{}} \begin{array}{@{}l@{}} {[ \rm {Fe{{( \rm {CN} )}}_6} ]}^{4 - } + {[ \rm {Fe( {\rm {TEOA}} )\rm OH} ]}^ - \rightleftharpoons \\ {[ \rm {Fe( {\rm {TEOA}} )\rm OH} ]}^{2 - } + {[ \rm {Fe{{( \rm {CN} )}}_6} ]}^{3 - } \end{array} \end{array}$$	1.34	[18]
A-S	Iron–chromium	$\rm F{e}^{2 + } + \rm C{r}^{3 + } \rightleftharpoons \rm C{r}^{2 + } + \rm F{e}^{3 + }$	1.18	[19]
A-S	Iron–manganese	$\rm F{e}^{2 + } + \rm M{n}^{3 + } \rightleftharpoons \rm M{n}^{2 + } + \rm F{e}^{3 + }$	0.79	[20]
A-S	Iron–vanadium	$\rm F{e}^{2 + } + \rm {V}^{3 + } \rightleftharpoons \rm {V}^{2 + } + \rm F{e}^{3 + }$	1.02	[21]
A-S	Iron–sulfur	$$\begin{array}{@{}l@{}} 2{[ \rm {Fe{{( \rm {CN} )}}_6} ]}^{4 - } + \rm {S}_2^{2 - } \rightleftharpoons \\ \rm 2{S}^{2 - } + 2{[ \rm {Fe{{( \rm {CN} )}}_6} ]}^{3 - } \end{array}$$	0.97	[22]
A-S	Iron–AQDS	$$\begin{array}{@{}l@{}} \rm 2FeS{O}_4 + \rm HS{O}_4{}^- + \mathrm{AQDS} + {\rm H}^+ \rightleftharpoons\\ \rm F{e}_2{( \rm {S{O}_4} )}_3 + \mathrm{AQDSH}_2 \end{array}$$	0.62	[23]

## FUNDAMENTAL PRINCIPLES FOR IRON-BASED REDOX ELECTROCHEMISTRY

**Figure 2.**
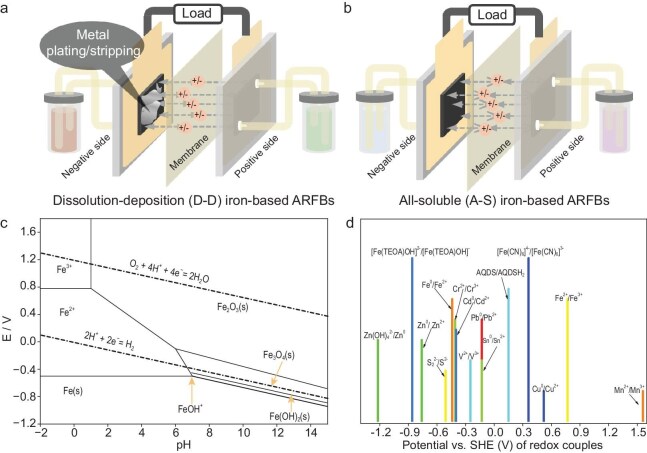
Schematic diagrams of (a) D-D iron-based ARFBs and (b) A-S iron-based ARFBs. (c) Pourbaix diagram for the Fe–H₂O system and (d) standard redox potentials of selected redox couples used in iron-based ARFBs.

Iron-based ARFBs rely on the redox chemistry of iron species to enable efficient and cost-effective energy storage. Understanding the fundamental electrochemical principles of these systems is crucial for optimizing their performance, stability and practical application. The fundamental principles governing these systems are rooted in the redox chemistry of iron, particularly the two primary redox couples: Fe^0^/Fe^2+^ and Fe^2+^/Fe^3+^. These redox reactions are influenced by various factors, including ligand coordination, pH and concentration, which affect solubility, reaction kinetics, energy density and overall cell efficiency. This section explores the electrochemical properties of these redox couples, the role of ligands in tuning redox potential, and strategies for maintaining stability during cycling. Finally, iron-based ARFBs are classified into two main categories—D-D ARFBs and A-S ARFBs—each of which can be further divided into all-iron and half-iron ARFBs, depending on the redox couples employed for the negative and positive electrodes (Fig. 2a and b).

### Fe^0^/Fe^2+^ redox couple

The Fe^0^/Fe^2+^ redox couple plays a critical role in deposition-type iron-based ARFBs, where metallic iron undergoes dissolution and deposition at the negative electrode. As shown in the Pourbaix diagram for the Fe–H₂O system (Fig. 2c), the standard redox potential of this reaction in aqueous solutions is approximately −0.44 V *vs.* the standard hydrogen electrode (SHE). The low potential and high volumetric capacity of deposited metals make high-energy-density flow batteries possible [24]. However, the practical reversibility and stability of this redox couple are governed by several interrelated electrochemical factors.

The iron plating and stripping process follows a two-electron transfer reaction (Equation 1):


(1) \begin{eqnarray*}
\rm F{e}^{2 + } + 2{\rm e}^ - \rightleftharpoons F{e}^0{\bf }\quad{{\mathrm{E}}}^{\mathrm{0}}={\mathrm{ - 0}}{\mathrm{.44 \,V }}\ \textit vs.\,{\mathrm{ SHE}}
\end{eqnarray*}

The kinetics and thermodynamics of this reaction are influenced by pH, complexation effects and mass transport limitations. In acidic conditions, Fe^2+^ remains highly soluble, minimizing passivation issues and unwanted oxidation. However, in near-neutral or alkaline conditions, the formation of hydroxyl complexes and iron oxides can hinder deposition and lead to surface passivation, reducing electrochemical activity. Additionally, the hydrogen evolution reaction (HER), a parasitic reaction of converting protons to H_2_ at the anode, competes with iron deposition in acidic media, reducing Coulombic efficiency (CE) and affecting long-term cycling stability. Electrodeposition of iron involves nucleation and growth mechanisms that depend on ion concentration, electrode surface properties and electrolyte composition. High Fe²⁺ concentrations can promote rapid nucleation but may also lead to dendrite formation, which poses a risk of short-circuiting and efficiency losses. Suppressing dendrite growth requires careful control of deposition conditions, such as current density, electrolyte additives and electrode surface modifications.

The presence of complexing agents like sulfate (SO_4_^2^^−^), chloride (Cl^−^) or organic ligands can significantly influence the redox potential, solubility and deposition behaviour of Fe^2+^ ions. Complexation can shift the redox equilibrium, alter charge transfer kinetics and stabilize iron species in solution, affecting the overall battery performance. Furthermore, since the Fe^0^/Fe^2+^ couple involves a phase transition between solid and dissolved states, the energy and power of these systems are no longer completely decoupled, unlike in fully soluble flow-battery chemistries. This intrinsic characteristic imposes limitations on operational flexibility and requires optimized flow-cell designs to balance energy storage capacity with stable cycling performance.

A comprehensive understanding of the fundamental electrochemistry governing iron plating and stripping—such as nucleation kinetics, interface charge transfer and competitive side reactions—is crucial for improving the efficiency and longevity of iron-based ARFBs. Addressing these challenges through electrolyte engineering, electrode surface modifications and advanced deposition control strategies will be key to realizing commercially viable high-performance iron-based flow batteries.

### Fe^2+^/Fe^3+^ redox couple

The Fe^2+^/Fe^3+^ redox couple, with a standard redox potential of approximately 0.77 V *vs.* SHE, is widely employed as the positive electrode reaction in all-iron and hybrid ARFBs. Its electrochemical behaviour is governed by fundamental factors such as pH, electrolyte composition and ligand coordination, which collectively impact redox potential, reaction kinetics and long-term stability.

The oxidation of Fe^2+^ to Fe^3+^ (Equation 2) is highly pH-dependent, as Fe^3+^ readily hydrolyzes at higher pH, forming insoluble hydroxides, e.g. Fe(OH)_3_, causing precipitation and capacity fading. Acidic electrolytes, typically sulfuric acid, prevent precipitation but can accelerate hydrogen evolution and material degradation.


(2) \begin{eqnarray*}
\rm F{e}^{2 + } \rightleftharpoons \rm F{e}^{3 + } + {\rm e}^ - {\bf }\quad{{\mathrm{E}}}^{\mathrm{0}}={\mathrm{0}}{\mathrm{.77 \,V }}\ \textit vs.\,{\mathrm{ SHE}}
\end{eqnarray*}

The coordination environment of Fe^2+^ and Fe^3+^ in solution plays a crucial role in determining their redox properties, solubility and stability. Inorganic ligands such as SO_4_^2^^−^ and Cl⁻ strongly interact with iron ions, shifting the redox potential and influencing the reaction kinetics. Sulfate-based coordination generally stabilizes Fe^2+^ in solution and improves redox reversibility, whereas chloride ligands can accelerate charge transfer but may also promote corrosion. Organic chelating agents, e.g. citrate, ethylenediaminetetraacetic acid (EDTA) or phosphonates, can further stabilize iron species in complexed forms, preventing precipitation and enabling broader pH operating windows, but may slow electron transfer kinetics. Strong coordination between cyanide (CN^−^) and Fe^2+^/Fe^3+^ enables the formation of non-toxic and non-corrosive ferricyanide [Fe(CN)_6_]^4^^−^^/3^^−^ compounds, which are widely studied in alkaline Fe–Zn ARFBs, neutral Fe–Sn ARFBs, alkaline all-iron ARFBs and Fe–sulfur (S) ARFBs. In 2015, Lin *et al.* reported the application of hydroxyl-substituted anthraquinones and [Fe(CN)_6_]^4^^−^^/3^^−^ in alkaline flow batteries to address serious cost, corrosion and safety issues in acidic flow batteries [25]. As shown in Equation 3, the standard potential of [Fe(CN)_6_]^4^^−^^/3^^−^ is + 0.36 V versus SHE, which is affected by the solution ionic strength, e.g. in the case of A-S all-iron ARFBs, the potential of the anodic [Fe(CN)_6_]^4^^−^^/3^^−^ is 0.48 V [26].


(3) \begin{eqnarray*}
&&{\left[ {\rm Fe{{\left( \rm {CN} \right)}}_6} \right]}^{4 - } \rightleftharpoons {\left[ \rm {Fe{{\left( \rm {CN} \right)}}_6} \right]}^{3 - } + \mathrm{e}^ - {\bf } \\
&&\quad{{\mathrm{E}}}^{\mathrm{0}}{\rm}= 0{\mathrm{.36 \,V }}\ \textit vs.\,{\mathrm{ SHE}}
\end{eqnarray*}

Increasing Fe^2+^/Fe^3+^ concentrations to enhance energy density presents additional challenges, including solubility constraints, increased electrolyte viscosity and mass transport limitations [27]. Ligand-assisted solubilization strategies can help mitigate these issues, though long-term cycling stability remains a challenge. Understanding the ligand coordination effects, charge transfer mechanisms and electrolyte interactions is critical for optimizing iron-based RFBs and developing hybrid systems with tailored properties.

### Design principles of iron-based ARFBs

The design of iron-based ARFBs is centered on selecting and coupling redox reactions to achieve an optimal balance of energy density, efficiency, stability and cost-effectiveness. Figure 2d shows the standard redox potentials (*vs.* SHE) of selected redox couples. The key iron redox couples—Fe^0^/Fe^2+^ and Fe^2+^/Fe^3+^—provide a foundation for different ARFB configurations, each offering distinct electrochemical characteristics and performance trade-offs. The Fe^0^/Fe^2+^ redox couple, with a standard potential of approximately −0.44 V *vs.* SHE, is commonly employed at the negative electrode due to its low potential and high volumetric capacity. However, its solid–liquid phase transition during charge–discharge cycles introduces challenges such as dendrite formation, hydrogen evolution and capacity degradation. In contrast, the Fe^2+^/Fe^3+^ redox couple, with a redox potential of ∼0.77 V *vs.* SHE, is fully soluble and often used at the positive electrode, enabling efficient charge transfer and avoiding solid-phase limitations.

Iron-based ARFBs can be broadly classified into two main categories: D-D ARFBs and A-S ARFBs. D-D ARFBs utilize the Fe^0^/Fe^2+^ redox couple at the negative electrode, where metallic iron is plated and stripped during charge–discharge cycles. This category includes all-iron ARFBs, which employ Fe^0^/Fe^2+^ at the negative electrode and Fe^2+^/Fe^3+^ at the positive electrode, and half-iron ARFBs, which pair Fe^0^/Fe^2+^ with a different redox reaction (e.g. vanadium-based or organic species) at the positive electrode to improve operational stability and mitigate phase transition issues. On the other hand, A-S ARFBs rely solely on liquid-phase reactions, avoiding solid-phase limitations. In this category, all-iron ARFBs use the Fe^2+^/Fe^3+^ redox couple at both electrodes, requiring ligand-stabilized iron complexes to prevent crossover and precipitation, while half-iron ARFBs couple Fe^2+^/Fe^3+^ with another soluble redox species, such as cerium (Ce) or vanadium, to enhance voltage and cycling performance. D-D ARFBs offer high energy density due to the solid-phase Fe storage and utilize abundant, low-cost materials. However, these systems face challenges related to dendrite formation, hydrogen evolution and mass transport limitations, which can reduce efficiency and cycle life. In contrast, A-S ARFBs provide better cycle stability and scalability by avoiding solid-phase transitions, yet they are often limited by the solubility of Fe^3+^ species, electrolyte viscosity and potential ligand degradation over prolonged cycling.

Among the various iron-based ARFB technologies, several promising configurations have emerged for practical applications. The all-iron flow battery (Fe^0^/Fe^2+^ || Fe^2+^/Fe^3+^) offers a high theoretical voltage and energy density, but further research is needed to address issues related to plating–stripping reversibility. Hybrid systems, such as Fe–V flow batteries (Fe^0^/Fe^2+^ || V^3+^/V^2+^), combine the cost advantages of iron with the stability of vanadium chemistry, offering a more balanced approach to performance and longevity. Meanwhile, Fe–organic flow batteries (Fe^2+^/Fe^3+^ || organic redox species) leverage redox-active organic molecules to enhance tunability and cycle life, while Fe–Ce flow batteries (Fe^2+^/Fe^3+^ || Ce^3+^/Ce^4+^) provide higher voltages (∼1.34 V) but require careful electrolyte management.

Future advancements in electrolyte formulation, electrode design and ligand coordination will be crucial for further optimizing iron-based ARFBs. By addressing key challenges such as dendrite suppression, hydrogen evolution mitigation and ligand stability, these systems have the potential to become a viable, scalable and cost-effective solution for long-duration energy storage, particularly in grid-scale applications.

## PROGRESS AND CHALLENGES OF IRON-BASED ARFBS

Based on their classification into D-D and A-S systems, substantial progress has been made in improving energy efficiency (EE), cycle life and scalability. Advances in electrolyte formulations, electrode materials and flow-cell designs have enhanced the stability and reversibility of iron redox reactions, addressing key challenges such as dendrite formation in Fe^0^/Fe^2+^ systems and ligand degradation in Fe^2+^/Fe^3+^ chemistries. Hybrid approaches, integrating iron with other redox-active species like vanadium, cerium and organic molecules, have further expanded the design space, enabling tailored voltage and operational stability. However, critical challenges remain, including hydrogen evolution, crossover effects and maintaining electrolyte stability over extended cycling. This section reviews the latest developments in iron-based ARFBs, highlighting key breakthroughs and ongoing research efforts to overcome existing limitations.

### D-D all-iron ARFBs

The development of D-D all-iron ARFBs can be traced back to 1981, when Hruska and Savinell first demonstrated the use of highly soluble iron-based chloride salts in an acidic flow battery system [28]. This early iteration, operating at 60 °C and achieved a CE of 90% and an EE of 50%, laid the foundation for the commercialization of acidic all-iron ARFBs [28]. These systems utilize ferric/metallic iron and ferric/ferrous redox couples in anolyte and catholyte, respectively, with the reactions described as follows (Equations 4–6):


(4) \begin{eqnarray*}
\mathrm{Negative}\!:&&\quad \rm F{e}^{2 + } + \rm 2{e}^ - \longleftrightarrow \rm F{e}^0{\bf }\\
&&\quad{{\mathrm{E}}}^{\mathrm{0}}\,{\mathrm{ = - 0}}{\mathrm{.44 \,V }}\,\textit vs.\,{\mathrm{ SHE}}
\end{eqnarray*}

(5) \begin{eqnarray*}
\mathrm{Positive}\!:&&\quad \rm F{e}^{2 + } \longleftrightarrow \rm F{e}^{3 + } + \rm {e}^ - {\bf }\\
&&\quad{{\mathrm{E}}}^{\mathrm{0}}\,{\mathrm{ = 0}}{\mathrm{.77 \,V }}\, \textit vs.\,{\mathrm{ SHE}}
\end{eqnarray*}

(6) \begin{eqnarray*}
\mathrm{Total}\!:&&\quad \rm 3F{e}^{2 + } \rightleftharpoons \rm F{e}^0 + \rm 2F{e}^{3 + }{\bf }\\
&&\quad{{\mathrm{E}}}^{\mathrm{0}}\,{\mathrm{ = 1}}\,{\mathrm{.21 \,V }}
\end{eqnarray*}

The use of multiple valence states of iron (Fe^0^, Fe^2+^ and Fe^3+^) prevents cross-contamination, as crossover ions can be rebalanced by remixing the electrolyte. Moreover, iron's abundant storage capacity and low-cost, eco-friendly nature make these systems highly attractive. Companies like ESS Tech, Inc. in the USA have made significant strides in developing and commercializing acidic all-iron ARFBs and the U.S. Advanced Research Projects Agency-Energy estimates that this iron-based flow battery would achieve an energy storage cost as low as $125 per kWh [29]. The ESS-designed first-generation full-scale acidic all-iron ARFB power module (S001) paired with ESS’s Gen I 50 kW/400 kWh Energy Warehouse systems demonstrates its ability to meet the needs of a wide range of grid operations users via connecting to the grid and responding in real time to grid controllers [30]. In 2019, ESS introduced the second-generation commercial power module (S200), which is fully bonded, addressing the electrolyte leakage issues that occurred in the S001 [30]. Most importantly, the cost of the S002 battery module is 50% less than that of the S100 module and can deliver up to five times the power of the S100 module [30]. In 2022, ESS provided an iron flow battery energy storage warehouse system for the Energy Storage Industries - Asia Pacific (ESIAP) company (Australia), and ESIAP announced that they are aiming to produce 200 MW/1.6 GWh of energy storage annually by the end of the year 2026. ESS and ESIAP have made great strides in commercializing the acidic all-iron ARFB to fill a gap of long-term energy storage application in the market.

Despite their advantages, D-D all-iron ARFBs face major challenges, primarily the poor reversibility of iron plating and stripping, which affects long-term stability. Another significant issue is HER, which competes with iron deposition at the anode:


(7) \begin{eqnarray*}
\mathrm{Anode}\!:&&\quad \rm 2{H}^ + + \rm 2{e}^ - = \rm {H}_2\\
&&\quad{{\mathrm{E}}}^{\mathrm{0}}\,{\mathrm{ = 0}}\,{\mathrm{.00 \,V }}\,\textit vs.\,{\mathrm{ SHE}}{\bf }
\end{eqnarray*}

This reaction competes with the intended iron deposition, leading to a reduction in CE and negatively affecting electrolyte stability [9]. As the HER process consumes hydronium ions, it can alter the pH of the electrolyte, further compromising the battery's performance. To combat this, extensive research has focused on electrolyte optimization, electrode modifications and improvements to the overall cell design. Adjusting the pH of the electrolyte and incorporating hydrogen collection and recovery devices have been identified as key strategies for mitigating HER and improving system efficiency.

Ultimately, while D-D all-iron ARFBs offer promising economic and environmental benefits, their commercial viability hinges on overcoming these challenges, particularly those related to reversibility and hydrogen evolution. Continued advancements in materials science and electrochemical engineering are crucial for addressing these issues and unlocking the full potential of iron-based flow batteries.

#### Electrolyte optimization

**Figure 3.**
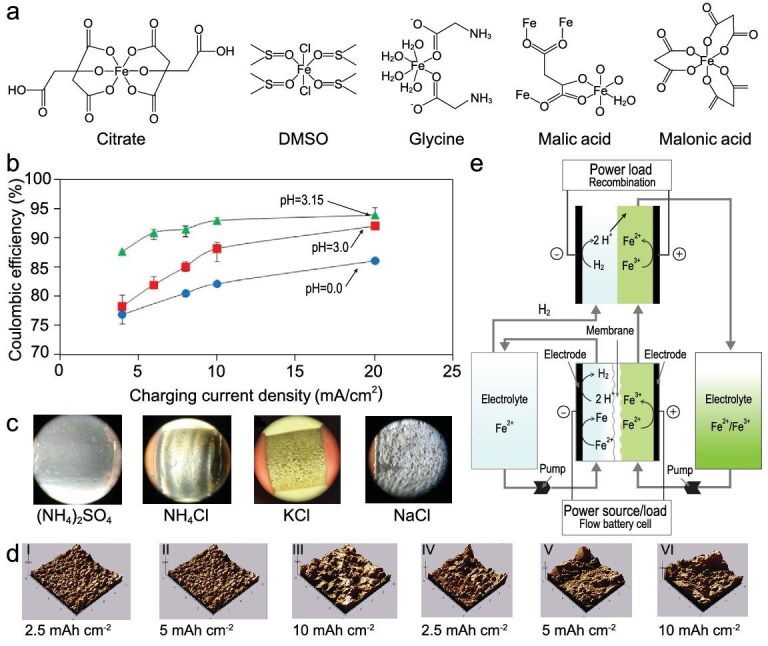
(a) Proposed structures for iron–ligand complexes for citrate, DMSO, glycine, malic acid and malonic acid. Reproduced with permission from Ref. [33]. Copyright 2014 IOP Publishing. (b) Effect of electrolyte pH on CE of iron electrodeposition and electrodissolution. Reproduced with permission from Ref. [39]. Copyright 2018 IOP Publishing. (c) The iron deposition on the copper rod electrodes with electrolytes containing 0.2 M FeCl_2_ and 1 M (NH_4_)_2_SO_4_, NH_4_Cl, KCl and NaCl at the current density of 20 mAh cm^–2^. Reproduced with permission from Ref. [41]. Copyright 2014 IOP Publishing. (d) Atomic force microscopy morphologies at the areal capacity of 2.5, 5 and 10 mAh cm^–2^ in (I–III) FeCl_2_–20% DMSO–80% H_2_O and (IV–VI) FeCl_2_–H_2_O. Reproduced with permission from Ref. [32]. Copyright 2022 Wiley-VCH GmbH. (e) Schematics of the acidic all iron ARFB with RC. Reproduced with permission from Ref. [9]. Copyright 2020 IOP Publishing.

Various methods have been explored to stabilize electrolyte pH and modify the solvation structure of ferrous ions, inhibiting HER and improving iron plating/stripping efficiency. Song *et al*. applied ligand field theory and first-principles calculations to screen out citric acid as an effective ligand, forming a stable Fe^2+^–citrate coordination structure [31]. This modification improved plating morphology and nearly eliminated HER, achieving 100% CE, nearly 100% capacity retention and 70% average EE over 300 cycles. Afterwards, they reported that the introduction of the polar solvent dimethyl sulfoxide (DMSO) into FeCl_2_-based anolytes yielded 75% EE at 30 mA cm^−2^ with negligible capacity fading over 200 cycles. [32]. The effects of pH value, supporting salts and additives on ARFB performance were further examined. Hawthorne and co-workers reported that increasing the pH from 1 to 2 significantly reduced HER, and high chloride concentration further suppressed it. Hawthorne *et al.* compared seven organic chemical ligands to stabilize ferric ions in the positive electrolyte (Fig. 3a), and glycine proved to be the most effective in stabilizing ferric ions without compromising redox kinetics [33].

Additionally, Na^+^ and Li^+^ cations were found to reduce iron deposition overpotential and HER, improving CE [34]. The use of indium chloride [35], zinc chloride [36] and ascorbic acid (AA) [37] also enhanced electrochemical performance by improving pH stability and suppressing unwanted side reactions. Temperature effects have also been studied extensively [34,38]. Raising the operating temperature to 80 °C reduced the plating potential by 150 mV, enhancing iron deposition more than HER, thus improving CE [39]. Jayathilake *et al.* found that AA can not only effectively inhibit HER in the pH range from 0 to 3, but also adjust electrode surface pH value (Fig. 3b) [39]. In the temperature range of 25–60°C, the improvement of CE is proportional to the increase in temperature. Under optimized conditions (pH = 3, 60°C), CE of all-iron ARFBs with AA can reach 97.9%. Furthermore, hydrochloric acid (HCl) supplementation minimized aging effects and increased capacity retention from 73% to 98% over 150 cycles [40]. Moreover, the additives that could regulate the iron plating and stripping have also attracted great attention. The sodium chloride (NaCl)-concentrated electrolyte presented the highest CE of 98% compared with the other salts, but the iron plating was not smooth (Fig. 3c) [41]. In addition, DMSO can induce the growth of iron preferentially on the Fe (110) crystal plane, which is beneficial for forming fine-grained nuclei (Fig. 3d) [32]. In 2024, Yang *et al.* proposed a highly soluble, polar and electron-donating additive, *N,N*-dimethylacetamide (DMAc), for operating all-iron flow batteries at low temperatures [42]. In an aqueous environment below −10°C, smooth and compact iron deposition was demonstrated on carbon felt (CF), indicating excellent Fe^2+^/Fe^0^ reversibility. At an ultra-low temperature of −20°C and 10 mA cm^−2^, the CE of the all-iron RFB reached 95%.

Despite extensive research efforts in electrolyte optimization, commercial all-iron flow batteries, according to the ESS Energy Center datasheet, still rely on a relatively simple FeCl_2_-based electrolyte composition, with an expected lifespan of 25 years. During the charging process, similar to laboratory-scale flow batteries, significant hydrogen evolution occurs at the negative electrode, leading to an increase in pH that can cause Fe^2+^ precipitation. Fe^3+^ will precipitate as ferric hydroxide at pH > 2, so any ferric ions that diffuse across the membrane will also precipitate both in the membrane and in the electrolyte. During standby, the deposited Fe metal is readily corroded, resulting in further hydrogen evolution. Good pH control is therefore critical in maintaining the efficient operation of the D-D iron flow cell. This usually involves the capture of any hydrogen generated during charging and by the iron corrosion reaction, and passing it through a rebalance cell to convert it back to protons. This will be detailed in a later section.

#### Modified electrodes

Electrode materials and structure have a significant impact on battery EE. Modifications such as metal catalyst decoration, heteroatom doping and functional carbon materials have been employed to enhance electrochemical performance [43]. For example, tungsten trioxide nanoparticles [44], graphene oxide [45] and Bermuda grass-derived nitrogen-doped carbon [46] have been used to increase active sites, enhance wettability and improve CE in all-iron ARFBs. Poly(pyrrole)/poly(4-styrenesulfonate)-coated porous carbon electrodes significantly extended the cycle life of all-iron ARFBs and effectively suppressed HER [47]. However, degradation of added functional materials can lead to electrolyte contamination, necessitating the use of intrinsic electrode modifications [48]. CF with abundant nanoscale carbon defects have been shown to enhance redox reversibility and iron deposition kinetics, yielding high CE (99%), high-power density (80 mW cm^−2^) and long cycling stability (>250 cycles) [48]. The high surface area anode electrode patented by ESS for the all-iron flow battery system shows dramatically improved round-trip efficiency (>70% at 200 mW cm^−2^) in the practical application [30].

In the Fe slurry flow cell, slurry electrodes serve as the negative half-cell, using solid particles to store and transport charge. A high particle volume fraction forms a conductive network for redox reactions [49]. This flowable electrode enables the decoupling of energy and power outputs when serving as the negative electrode in all-iron ARFBs. Petek *et al.* selected multi-walled carbon nanotubes for slurry electrodes in all-iron flow batteries, achieving minimal iron deposition on the current collector [49]. This enhanced conductivity supports directional iron plating. However, despite promising laboratory-scale performance, 10-cell stacks exhibited only 50% efficiency at 50 mA cm⁻² due to hydrogen evolution and slurry plugging, highlighting challenges in scaling up this electrode configuration for all-iron aqueous RFBs [50].

#### Membrane selection

The key function of the membrane in the ARFB is to ensure that the anolyte and catholyte remain physically separated and unmixed, and to provide a pathway for selective ion exchange between the two sides of the battery [51]. Polyvinyl alcohol (PVA)-based membranes have been used in the direct methanol fuel cell [52]. Inspired by this, Sinclair *et al.* prepared a PVA hydrogel composite Daramic membrane, which can significantly reduce hydraulic permeability and ionic crossover, which is a promising membrane candidate for all-iron ARFBs [53].

#### System improvement

The electrolyte imbalances issue resulting from HER can be resolved by using an appropriate rebalancing system [54]. In 2020, Noack *et al.* developed a recombination cell (RC) to convert hydrogen gas back into protons, thereby reducing irreversible capacity loss (Fig. 3e) [9]. By integrating a platinum on carbon catalyst-coated Nafion membrane, hydrogen generated at the negative side is oxidized to protons in the RC, restoring electrolyte balance and improving battery longevity. Optimized all-iron RFBs with this system achieved 70% EE at 12.5 mA cm^−2^. In commercial all-iron ARFBs that are being installed around the world, ESS employs so-called proton pumps to manage the state of charge and electrolyte pH imbalance. When applying the proton pump, the ESS all-iron flow battery system has been shown to cycle up to 1000 times without significant performance loss or capacity degradation. Most importantly, the protons are driven directly back into the catholyte, thus maintaining the electrolyte pH within a stable range [30].

D-D all-iron ARFBs present a cost-effective and environmentally friendly alternative to conventional flow batteries. However, overcoming challenges related to iron plating reversibility, HER suppression and electrolyte stability is crucial for commercialization. Advances in electrolyte formulation, electrode design and system architecture continue to push the boundaries of efficiency and durability, bringing these promising energy storage solutions closer to widespread adoption.

### D-D half-iron ARFBs

D-D half-iron ARFBs represent a promising alternative to traditional all-iron systems by incorporating metal plating and stripping processes of non-iron metals on the negative side. Pairing Fe^2+^/Fe^3+^ with metals like zinc or tin opens up the potential for developing low-cost, environmentally friendly flow battery systems by leveraging the unique redox potentials of different metal pairs. For example, Fe–Zn systems exhibit a wide range of pH compatibility, with the alkaline Fe–Zn ARFB achieving a high overall cell voltage of up to 1.58 V. Nevertheless, optimizing redox potentials, electrochemical kinetics and system design remains challenging. This section explores strategies for improving electrolytes, electrodes, membranes and battery structures. A key concern is ion diffusion across the membrane, causing irreversible capacity loss, highlighting the need for high membrane selectivity in long-term operation.

### Fe–Zn ARFBs

As one of the most abundant elements in the Earth's crust, zinc has been a focal point of current research with high specific energy density and low redox potential. Fe–Zn ARFBs present advantages such as low cost, safety and environmental friendliness, making them promising long-term alternatives to all-vanadium ARFBs. In 2015, Gong *et al.* developed a novel dual-membrane, triple-electrolyte Fe–Zn ARFB, incorporating alkaline zinc ions at the anode and acidic iron ions at the cathode [55]. This configuration maintained a CE of 99.9% at a current density of 80 mA cm^−2^, with costs significantly below the 2023 U.S. Department of Energy's target of $150 kWh^−1^. However, issues such as ion crossover and the growth of zinc dendrites still severely hinder its development, as is the ability of the dual membrane to maintain separation of the alkaline and acidic half-cell solutions over the life of the battery.

Ongoing research categorizes Fe–Zn ARFBs into acidic, alkaline and neutral types. In an acidic Fe–Zn ARFB system, the Fe^2+^/Fe^3+^ redox pair possesses higher solubility and faster kinetics in acidic solutions, which is favourable for improving the battery performance [56]. However, the low pH environment influences zinc deposition and iron hydrolysis, making HER a significant issue. Alkaline Fe–Zn ARFBs employ the redox [Fe(CN)_6_]^4−^/[Fe(CN)_6_]^3−^ pair with Zn^2+^/Zn^0^, achieving a high cell voltage of 1.58 V (Equations 8–10).


(8) \begin{eqnarray*}
&&\mathrm{Negative}\!:\quad Zn{\left( {OH} \right)}_4^{\,2 - } + \ 2{e}^ - \longleftrightarrow Z{n}^0+ \ 4O{H}^ - {\bf }\\
&&\quad\qquad\qquad{{\mathrm{E}}}^{\mathrm{0}} ={\mathrm{ - 1}}{\mathrm{.22 \,V }}\,\textit vs.\,{\mathrm{ SHE}}
\end{eqnarray*}

(9) \begin{eqnarray*}
&&\!\!\!\mathrm{Positive}\!:\!\!\quad 2{\left[ {Fe{{\left( {CN} \right)}}_6} \right]}^{4 - }\! \longleftrightarrow\! 2{\left[ {Fe{{\left( {CN} \right)}}_6} \right]}^{3 - } \!+ 2{\rm e}^ - {\bf }\\
&&\qquad\qquad\ {{\mathrm{E}}}^{\mathrm{0}}\,{\mathrm{ = 0}}\,{\mathrm{.36 \,V }}\,\textit vs.\,{\mathrm{ SHE}}\!\!\!\!
\end{eqnarray*}

(10) \begin{eqnarray*}
&&\!\!\!\mathrm{Total}\!:\quad 2{\left[ {Fe{{\left( {CN} \right)}}_6} \right]}^{4 - } + Zn{\left( {OH} \right)}_4^{\,2 - } \rightleftharpoons {\bf }\\
&&\quad \qquad 2{\left[ \mathrm{Fe{{\left( \mathrm{CN} \right)}}_6} \right]}^{3 - } + \ \rm Z{\rm n}^0 + \ 4O{\rm H}^ -\\
&&\quad \qquad {{\mathrm{E}}}^{\mathrm{0}} = {\mathrm{ 1}}{\mathrm{.58 \,V }}
\end{eqnarray*}

**Figure 4.**
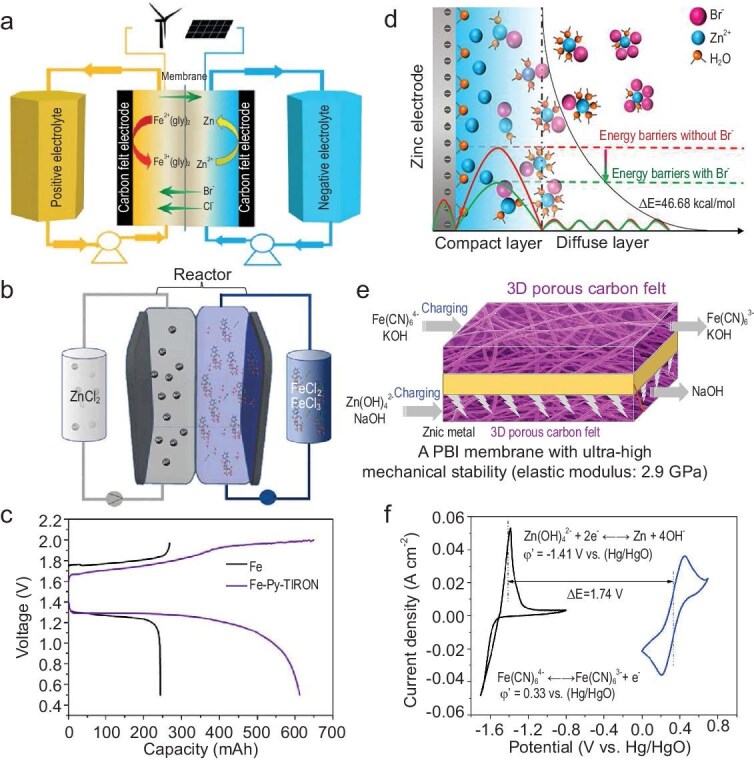
(a) The schematic diagram of neutral Fe-Zn ARFB. Reproduced with permission from Ref. [12]. Copyright 2017 Wiley-VCH GmbH. (b) Schematic diagram of an Fe–Zn ARFB with TRION. (c) The capacity–voltage profiles of (b) at a current density of 10 mA cm^–2^. Reproduced with permission from Ref. [58]. Copyright 2024 Elsevier Ltd. (d) Schematic of Zn^2+^ plating progress at the double layer region on the electrode surface. Reproduced with permission from Ref. [64]. Copyright 2022 Elsevier B.V. (e) Schematic of introjecting a PBI membrane with ultra-high mechanical stability and a 3D porous CF for an alkaline Fe–Zn ARFB. (f) CV curves of redox couples in (e). Reproduced with permission from Ref. [66]. Copyright 2018 Elsevier Inc.

Unlike acidic systems, alkaline Fe–Zn ARFBs are not plagued by severe HER but still suffer from zinc dendrite formation, reducing cycle life. Neutral Fe–Zn ARFBs mitigate the corrosion and environmental concerns of acidic and alkaline batteries. Xie *et al.* introduced the first neutral Fe–Zn ARFB in 2017 (Fig. 4a) [12]. However, these systems suffer from the low solubility of [Fe(CN)_6_]^4^^−^^/3^^−^ and the Zn_2_Fe(CN)_6_ precipitate induced by the crossover of Zn^2+^ to catholyte. To address these issues, advancements in electrolyte additives, membrane modifications and electrode improvements have been reported.

#### Electrolyte optimization

To alleviate HER in acidic Fe–Zn ARFBs, Xie *et al.* proposed using an acetate/sodium acetate anhydrous buffer to suppress HER while improving zinc plating and stripping [10]. Subsequent studies introduced chloride-based additives to enhance the reaction kinetics [57]. Tippayamalee *et al.* presented a dual-ligand system incorporating pyridine (Py) and disodium 4,5-dihydroxy-1,3-benzenedisulfonate (TIRON), effectively mitigating iron hydrolysis and improving electrochemical stability (Fig. 4b and c) [58]. Zhi *et al.* identified EDTA tetrasodium as a chelating agent, effectively improving zinc solvation and extending service life to 2300 h in alkaline Fe–Zn ARFBs [59]. Other research has explored urea as a zinc salt-complexing agent to suppress Zn dendrites [60]. To address electrolyte migration, Liu *et al.* studied water transport across membranes, finding that glucose was the most effective additive for inhibiting water migration, thanks to the polymeric form of the aldehyde group in an alkaline solution [61]. To combat the ion crossover issues in neutral Fe–Zn ARFBs, the introduction of sodium citrate in the cathode electrolyte to coordinate with Zn^2+^ enables the formation of a zinc citrate complex. This complex interacts with the sulfonic acid group (-SO_3_^–^) on the cation exchange membrane (SPEEK), mitigating Zn^2+^ crossover and preventing precipitation via the Donnan repulsion effect [62]. Despite these advancements, neutral Fe–Zn ARFBs suffer from low ionic conductivity, leading to high internal resistance and reduced EE. Additionally, uncontrolled dendrite growth from continuous zinc deposition and stripping presents a significant challenge. In response, Yang *et al.* used nicotinamide as an additive in the neutral ZnCl_2_ anode electrolyte, altering the solvation structure of zinc, effectively suppressing the tip effect of zinc deposition [63]. Furthermore, bromide ions can stabilize zinc ions through complexation, enhancing EE and cycling stability in neutral Fe–Zn ARFBs (Fig. 4d) [64].

#### Electrode modification

Polyvinylidene fluoride compressed electrode materials have exhibited high cycling performance (CE maintained above 96% after 50 cycles and EE of 61%) in acidic Fe–Zn ARFBs [65]. High selectivity and a 3D porous CF electrode (Fig. 4e and f) have been designed to render alkaline Fe–Zn ARFBs able to achieve excellent CE (99.97%) and EE (88.07%) [66]. In addition, modification of the electrode through element doping has become an area of significant interest for researchers. Zhu *et al.* have developed boron-doped CF electrodes with abundant electron-deficient sites and defects to enhance the diffusion of Zn(OH)_4_^2–^ on the electrode surface, finally constructing high-performance alkaline Fe–Zn ARFBs [67].

#### Membrane improvement

The development of an anion exchange membrane (AEM) with superior mechanical strength and alkali corrosion resistance contributes to eliminating the crossover of active ions in acidic Fe–Zn ARFBs [68]. For an alkaline battery system, the method to improve the performance involves developing polybenzimidazole (PBI) membranes (Fig. 4e) [66]. Montmorillonite-based composite membranes have also demonstrated efficacy in regulating zinc deposition, reducing dendrite-induced damage [69]. To improve performance and cost-effectiveness of neutral Fe–Zn ARFBs, Chang *et al*. developed a K^+^-doped sulfonated poly(ether ether ketone) membrane, which reduced the cost of ion exchange membranes while ensuring good proton conductivity and low internal resistance [70].

With ongoing advancements in materials and structural design, Fe–Zn ARFBs have the potential to offer a promising, cost-effective and resource-abundant energy storage solution for the future, although the issue of ion transfer across the membrane will always lead to irreversible capacity loss with all flow cell chemistries employing different elements in the two half-cells. The design of membranes that can completely separate anode and cathode substances and conduct ions smoothly is an effective strategy to address ion crossover through investigating the chemistry and microstructure associated with ion transport channels. Currently, adamantane-containing poly(ether ketone) AEMs, terphenyl AEMs, poly(sulfone)-based AEMs etc. have been reported in other RFB systems, providing novel ideas for the future development direction of membrane modification in Fe–Zn ARFBs [71].

#### Commercial status

With the continuous exploration of the three core components of the flow battery and the vigorous development of large-scale power stations, the demonstration platform of Fe–Zn ARFBs has been installed in several countries. In 2013, the BlueSky Energy company (Australia) established a 64 kw Fe–Zn RFB system in Europe. Subsequently, the Ontario Independent Electricity System Operator partnered with ViZn Energy Systems Inc. to build a 2 MW/6 MWh Fe–Zn ARFB system to provide energy storage services to the Ontario grid. In China, Jinsheng New Energy Technology Co., Ltd. developed and commissioned a 10 kW/10 kWh Fe–Zn ARFB energy system [72]. The establishment of the above-mentioned demonstration platforms proves that Fe–Zn ARFBs are an efficient and stable large-scale energy storage technology. However, many challenges need to be combated to achieve the level for commercialization and industrialization.

### Other D-D half-iron ARFBs

In addition to zinc, several other iron-based ARFB systems have been developed, incorporating various metal (e.g. tin, lead, cadmium and copper) plating/stripping reactions. Tin (Sn) is a widely used metal known for its excellent ductility, good thermal and electrical conductivity, and superior kinetic properties. While E^0^ = −0.13 V *vs.* SHE (Equation 11) suggests that Sn deposition occurs near the HER potential, the high hydrogen overpotential of tin helps suppress HER, making it a strong candidate for the negative electrode in iron-based ARFBs.


(11) \begin{eqnarray*}
\mathrm{Negative}\!:&&\quad \rm S{n}^{2 + } + \rm 2{e}^ - \longleftrightarrow\ \rm S{n}^0{\bf }\\
&&\quad{{\mathrm{E}}}^{\mathrm{0}}\,{\mathrm{ = - 0}}{\mathrm{.13 \,V }}\,\textit vs.\,{\mathrm{ SHE}}
\end{eqnarray*}

(12) \begin{eqnarray*}
\mathrm{Positive}\!:&&\quad \rm F{e}^{2 + } \longleftrightarrow \rm F{e}^{3 + } + \rm {e}^ - {\bf }\\
&&\quad{{\mathrm{E}}}^{\mathrm{0}}={\mathrm{ 0}}{\mathrm{.77 \,V }}\,\textit vs.\,{\mathrm{ SHE}}
\end{eqnarray*}

(13) \begin{eqnarray*}
\\ &&\mathrm{Total}\!:\quad 2F{e}^{2 + } + \ S{n}^{2 + } \rightleftharpoons S{n}^0 + \ 2F{e}^{3 + }{\bf } \\
&&\qquad\qquad{{\mathrm{E}}}^{\mathrm{0}}= {\mathrm{ 0}}{\mathrm{.90 \,V }}
\end{eqnarray*}

**Figure 5.**
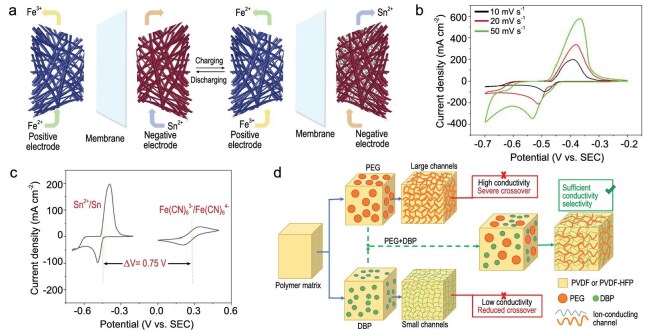
(a) Schematic diagram of the working progress of the Fe–Sn ARFB. Reproduced with permission from Ref. [13]. Copyright 2018 Elsevier B.V. (b) CV curves of Sn anode at different scan rates. (c) CV curves of cathode and anode sides for Fe–Sn ARFB at the current density of 10 mV s^−1^. Reproduced with permission from Ref. [14]. Copyright 2021 IOP Publishing. (d) Schematic diagram of the method applying multiple templates by using different combinations of polyethylene glycol (PEG) and diphthalate (DBP) to achieve adjustable membrane performance in Fe–Pb ARFB. Reproduced with permission from Ref. [77]. Copyright 2024 OAE Publishing Inc.

The low anisotropy of tin contributes to its outstanding electrochemical performance, including uniform electrochemical reactions and consistent deposition and corrosion behaviours. Zhou *et al.* developed an acidic Fe–Sn ARFB using FeCl_3_/FeCl_2_ as the catholyte (Fig. 5a), achieving an average CE of 99% and EE close to 80% over 700 cycles at 200 mA cm^−2^ [13].

Despite the high HER overpotential of Sn, hydrogen evolution in a strongly acidic environment remains an inevitable issue, alongside uncontrolled metal deposition. To address this, Zhou *et al.* proposed engineering modifications to Fe–Sn ARFBs and found that serpentine flow paths and low flow rates significantly improved the deposition morphology of tin, leading to more uniform electrodeposition due to limited active material transport [73]. Enhancing battery longevity and mitigating capacity decay remain critical goals in improving system design [74]. However, the above-mentioned acidic Fe–Sn ARFBs face drawbacks, including corrosion of internal components, environmental concerns and unavoidable HER in acidic electrolytes. Developing a neutral Fe–Sn ARFB presents a promising alternative. In 2021, Chen *et al.* introduced a dendrite-free neutral Fe–Sn ARFB with an overall cell voltage of 0.75 V (Equation 14 and Fig. 5b and c), achieving over 97% CE and 80% EE after 120 cycles at a current density of 10 mA cm^−2^ while retaining more than 60% of its initial capacity [14]. These advancements highlight the strong potential of Fe–Sn ARFBs as next-generation energy storage solutions with good electrochemical performance and cost-effectiveness.


(14) \begin{eqnarray*}
&&{\mathrm{2}}{\left[ {{\mathrm{Fe}}{{\left( {{\mathrm{CN}}} \right)}}_{\mathrm{6}}} \right]}^{{\mathrm{4}} - }{\mathrm{ +\, S}}{{\mathrm{n}}}^{{\mathrm{2 + }}} \rightleftharpoons {2\left[ {{\mathrm{Fe}}{{\left( {{\mathrm{CN}}} \right)}}_{\mathrm{6}}} \right]}^{{\mathrm{3}} - }{\rm +\, S}{{\mathrm{n}}}^{\mathrm{0}}\\
&&\quad\quad{{\mathrm{E}}}^{\mathrm{0}}\,{\mathrm{ = 0}}{\mathrm{.75\ V}}
\end{eqnarray*}

Iron-lead (Fe–Pb) ARFBs, proposed by Zeng *et al.* in 2017 (Equations 15–17), improve kinetics and reduce HER and Coulombic losses compared with acidic all-iron ARFBs [15].


(15) \begin{eqnarray*}
\mathrm{Negative}\!:&&\quad \rm P{b}^{2 + } + \rm 2{e}^ - \longleftrightarrow \rm P{b}^0{\bf }\\
&&\quad{{\mathrm{E}}}^{\mathrm{0}}\,{\mathrm{ = }} - {\mathrm{0}}{\mathrm{.13 \,V }}\,\textit vs.\,{\mathrm{ SHE}}
\end{eqnarray*}

(16) \begin{eqnarray*}
\mathrm{Positive}\!:&&\quad \rm F{e}^{2 + } \longleftrightarrow \rm F{e}^{3 + } + \rm {e}^ - {\bf }\\
&&\quad\quad{{\mathrm{E}}}^{\mathrm{0}}\,{\mathrm{ = 0}}{\mathrm{.77\ V \,\textit{vs}}}.\,{\mathrm{ SHE}}
\end{eqnarray*}

(17) \begin{eqnarray*}
&&\mathrm{Total}\!:\quad \rm 2F{e}^{2 + } + \ \rm P{b}^{2 + } \rightleftharpoons \rm P{b}^0 + \ \rm 2F{e}^{3 + }{\bf }\\
&&\qquad\qquad{{\mathrm{E}}}^{\mathrm{0}}\,{\mathrm{ = 0}}{\mathrm{.90 \,V }}
\end{eqnarray*}

Using methanesulfonic acid (MSA) as the supporting acid medium provides high solubility and strong kinetics for both the positive and negative redox couples. Additionally, in this strong acid system, the internal resistance of the Fe–Pb ARFB was significantly reduced compared with the 4.2 Ω cm^–2^ of the all-iron RFB. Electrode modifications, such as 3D-structured lead electrodes with acetylene black (ACET), enhanced porosity and conductivity but showed performance trade-offs at high ACET content [75]. In 2024, Jiang *et al.* further optimized the graphite plate cathode via the electrochemical corrosion method, achieving a 77.88% VE and 77.13% EE at 40 mA cm^−2^ [76]. Membrane advancements also contributed to performance improvements. Zhang *et al*. developed a porous non-ionic polymer membrane with high proton conductivity (43.5 mS cm^−1^), inhibiting the crossover diffusion of iron ions (Fig. 5d). An Fe–Pb ARFB using this membrane maintained a CE of over 99.9% at 50 mA cm^−2^ and 98.1% after 200 cycles at 20 mA cm^−2^ [77]. Its competitive active material cost ($36.35 kWh⁻¹) further enhances feasibility.

Iron–cadmium (Fe–Cd) ARFBs offer projected costs as low as $10 kWh^−1^, but ion concentration gradients cause cross-contamination of the two half-cell solutions. Zeng *et al.* mitigated this by mixing catholyte and anolyte, achieving ∼80% EE and capacity retention of 99.87% per cycle [16]. While mixing the two elements in each half-cell eliminates cross contamination, mixed electrolyte systems require the use of twice the amount of each metal ion, half of which remains inactive, while doubling the cost of the electrolytes. Rayon- or polyacrylonitrile-modified CFs were studied by Zhang and co-workers [78]. The former has sufficient oxygen functional groups and catalytic effects, so it exhibits slower capacity decay, and the latter has higher voltage efficiency (VE) and EE due to its high degree of graphitization. Cost-effective all-copper (Cu) ARFBs, initially explored by Sanz *et al.* in 2014, suffered from low battery potential [79]. Kabtamu *et al.* later introduced a low-cost Fe–Cu ARFB incorporating heat-treated graphite felt (GF)/carbon paper and bismuth (Bi^3+^) additives to improve the reversibility of Cu^+^/Cu^0^, achieving a CE of 89.18% and EE of 35.24% at 20 mA cm^−2^ [17].

### A-S all-iron ARFBs

Among iron-based ARFBs, A-S all-iron ARFBs stand out by utilizing soluble iron species for both the anolyte and catholyte (Equations 18–20; TEOA being triethanolamine), offering a mechanism advantage comparable to all-vanadium ARFBs [18]. Unlike D-D all-iron ARFBs, A-S all-iron ARFBs maintain a nearly constant redox species concentration during charge–discharge cycles. This is achieved by replacing water molecules around the iron ion with organic ligands to form Fe^2+^/Fe^3+^–organic redox pairs, avoiding the poor reversibility of the Fe^2+^/Fe^0^ redox couple. The unique A-S ARFB design allows for independent scaling of energy capacity and output power by adjusting electrolyte concentration and volume. Benefiting from higher electrolyte utilization, A-S all-iron ARFBs perform well in improving EE, reducing waste and rendering the system more cost-effective. Nevertheless, battery performance still has significant room for improvement by optimizing the electrolyte and exploring new electrode materials.


(18) \begin{eqnarray*}
\mathrm{Negative}\!:\quad\!\!\!\! &&{\left[ \mathrm{Fe\left( {\mathrm{TEOA}} \right)\rm OH} \right]}^ - + \mathrm{e}^ - \!\longleftrightarrow\! {\bf }\\
&&\quad{\left[ {\rm Fe\left( {\mathrm{TEOA}} \right)\rm OH} \right]}^{2 -}\\
&&{{\mathrm{E}}}^{\mathrm{0}}\ {\mathrm{ = }} - {\mathrm{0}}{\mathrm{.86 \,V }}\,\textit vs.\,{\mathrm{ SHE }}
\end{eqnarray*}

(19) \begin{eqnarray*}
&&\mathrm{Positive}\!:\quad {\left[ {\rm Fe{{\left( {\rm CN} \right)}}_6} \right]}^{4 -} \longleftrightarrow {\left[ {\rm Fe{{\left( {\rm CN} \right)}}_6} \right]}^{3 -}+ \ {e}^ - {\bf } \\
&&\quad\qquad\quad{{\mathrm{E}}}^{\mathrm{0}}\ {\mathrm{ = 0}}{\mathrm{.48 \,V }}\,\textit vs.\,{\mathrm{ SHE}}
\end{eqnarray*}

(20) \begin{eqnarray*}
&&\mathrm{Total}\!:\quad {\left[ {\rm Fe{{\left( {\rm CN} \right)}}_6} \right]}^{4 -} + \ {\left[ {\rm Fe\left( {\mathrm{TEOA}} \right)\rm OH} \right]}^ - \rightleftharpoons {\bf } \\
&&\quad\,\,\quad\qquad {\left[ {\rm Fe{{\left( {\rm CN} \right)}}_6}\right]}^{3 -}+ {\left[ {\rm Fe\left( {\mathrm{TEOA}} \right)\rm OH} \right]}^{2 -} \\
&&\quad\quad\quad\quad{{\mathrm{E}}}^{\mathrm{0}}= 1{\mathrm{.34 \,V }}
\end{eqnarray*}

Research in this field primarily focuses on ligand design. Anolytes and catholytes used in A-S all-iron ARFBs are composed of water-soluble iron salts and supporting electrolytes, which can be categorized into alkaline and near-neutral systems. In neutral or alkaline conditions, Fe–ligand must have a formation equilibrium and stability constant higher than Fe(OH)₃ to prevent iron hydroxide precipitation [26]. To enhance stability, multidentate organic ligands are commonly explored. Ferrocyanide and water-soluble carboxylic acid-modified ferrocyanide salts are commonly employed as catholytes due to their positive redox potentials and exceptional stability over a wide pH range. The anolyte ligands used in A-S all-iron-ARFBs can be seen in Table 2. In strongly alkaline conditions, amino alcohol represents a commonly used class of anolyte ligands. Yan *et al.* first employed triethanolamine (TEOA) as the anolyte ligand, paired with a ferrocyanide-based catholyte, to develop an A-S all-iron RFB. This system achieved a voltage of 1.34 V and demonstrated outstanding performance [18]. To address the dissociation of TEOA, research on A-S all-iron ARFBs has focused on identifying more suitable anode ligands. Our previous studies summarized the recent efforts that have explored new ligands such as 3-[bis(2-hydroxyethyl)amino]-2-hydroxypropanesulfonic acid (DIPSO), 2,2-bis(hydroxymethyl)-2,2′,2′-nitrilotriethanol (Bis-Tris), sodium gluconate (Gluc-), TEOA–2-methylimidazole (TEOA–MM), sodium 3,3′,3′′,3′′′-(ethane-1,2-diylbis(azanetriyl))tetrakis(2-hydroxypropane-1-sulfonate) (EDTS), Na[iron(III)-*N,N*′-ethylene-bis(*o*-hydroxyphenylglycine)] (Fe^III^-racEDDHA), nitrilotri-(methylphosphonic acid) (NTMPA) and composite ligand [26]. Additionally, studies have demonstrated that A-S all-iron ARFBs also can operate in near-neutral environments [80], further broadening their range of applications.

**Table 2.** A summary of basic properties for A-S all-iron ARFBs with different electrolytes. Reproduced with permission from Ref. [26]. Copyright 2025 Elsevier B.V.

Anolyte		Catholyte			A-S all-iron ARFB performance				
Iron salts	Ligand	compound	Overall cell voltage (V)	Current density (mA cm^−^^2^)	CE (%)	VE (%)	EE (%)	Cycles	Capacity retention (%)
FeCl_3_	TEOA	Na_4_Fe(CN)_6_	1.34	40	93	78	73	110	
Fe_2_(SO_4_)_3_	TEOA	K_4_Fe(CN)_6_		40	93	78	72	50	
Fe_2_(SO_4_)_3_	DIPSO	Na_4_Fe(CN)_6_	1.37	80	99.5		68.5	100	88
Fe_2_(SO_4_)_3_	Bis-Tris	Na_4_Fe(CN)_6_ + K_3_Fe(CN)_6_	1.43	80	99.8		72.8	250	∼99.9
FeCl_3_	Gluc^−^	Na_4_Fe(CN)_6_	1.19	80	>99		∼83	950	83.74
FeCl_3_	TEOA−MM	K_4_Fe(CN)_6_	1.373	80	98.5	81.8	80.5	1400	∼100
FeCl_3_	EDTS	K_4_Fe(CN)_6_	1.25	80	99.93	73.59	73.54	3000	96.08
Na[Fe^III^-racEDDHA]		Na_4_Fe(CN)_6_	0.848	20	∼100	68		75	
FeCl_3_	NTMPA	K_4_Fe(CN)_6_ + Na_4_Fe(CN)_6_	∼0.6	20	100		87	1000	96
		Fe–Dcbpy/CN	∼1					100	

Dcbpy, dicarboxylic bipyridine.

### A-S half-iron ARFBs

A-S half-iron ARFBs utilize iron ions as active materials in the anolyte, paired with other metal ions or organic/inorganic redox couples. A-S half-iron ARFBs mitigate CE and EE losses that arise from the poor reversibility of metal plating/stripping. However, A-S half-iron ARFBs face several challenges that hinder their large-scale implementation. One major limitation is the low solubility of redox-active species, which restricts energy density. Additionally, sluggish redox kinetics reduce power density and overall efficiency, necessitating the development of electrocatalysts and electrolyte modifications. Furthermore, the HER in acidic conditions competes with the redox reactions, leading to self-discharge and electrolyte degradation. These challenges are particularly pronounced in Fe–Cr, Fe–manganese (Mn) and Fe–S ARFBs. Recent research has focused on enhancing the performance of A-S half-iron ARFBs through electrode modification, electrolyte optimization and the development of alternative redox pairs.

### Fe–Cr ARFBs

Fe–Cr ARFBs utilize Fe^2+^/Fe^3+^ and Cr^2+^/Cr^3+^ as redox couples, producing a standard cell voltage of 1.18 V (Equation 23) [19], with an estimated cost of just $9.4 kWh^−1^ [81].


(21) \begin{eqnarray*}
\mathrm{Negative}\!:&&\quad \rm C{r}^{3 + } + \rm {e}^ - \longleftrightarrow \rm C{r}^{2 + }{\bf }\\
&&\quad{{\mathrm{E}}}^{\mathrm{0}}\,{\mathrm{ = }} - {\mathrm{0}}{\mathrm{.41 \,V }}\,\textit vs.\,{\mathrm{ SHE}}
\end{eqnarray*}

(22) \begin{eqnarray*}
\\\mathrm{Positive}\!:&&\quad \rm F{e}^{2 + } \longleftrightarrow \rm F{e}^{3 + } + \ {\rm e}^ - {\bf }\\
&&\quad{{\mathrm{E}}}^{\mathrm{0}}={\mathrm{ 0}}{\mathrm{.77 \,V }}\,\textit vs.\,{\mathrm{ SHE}}
\end{eqnarray*}

(23) \begin{eqnarray*}
&&\mathrm{Total}\!:\quad F{e}^{2 + } + C{r}^{3 + } \rightleftharpoons C{r}^{2 + } + \ F{e}^{3 + }{\bf }\\
&&\qquad\quad\quad{{\mathrm{E}}}^{\mathrm{0}}={\mathrm{ 1}}{\mathrm{.18 \,V }}
\end{eqnarray*}

From a 10 kW Fe–Cr flow battery system developed by the Sumitomo Electric Company (Japan) to the megawatt Fe–Cr flow battery energy storage systems that the China State Power Investment Group have successfully installed, Fe–Cr ARFBs have been developed for over 50 years, which hold great potential to reach commercialization level [82]. Although they offer cost advantages and high safety, their further development is hindered by the slow kinetics of the Cr^2+^/Cr^3+^ redox couple and HER on the negative side. To combat these issues, researchers have explored solutions, including electrode modification, electrolyte optimization, membrane improvement and flow battery design.

#### Electrode modification

**Figure 6.**
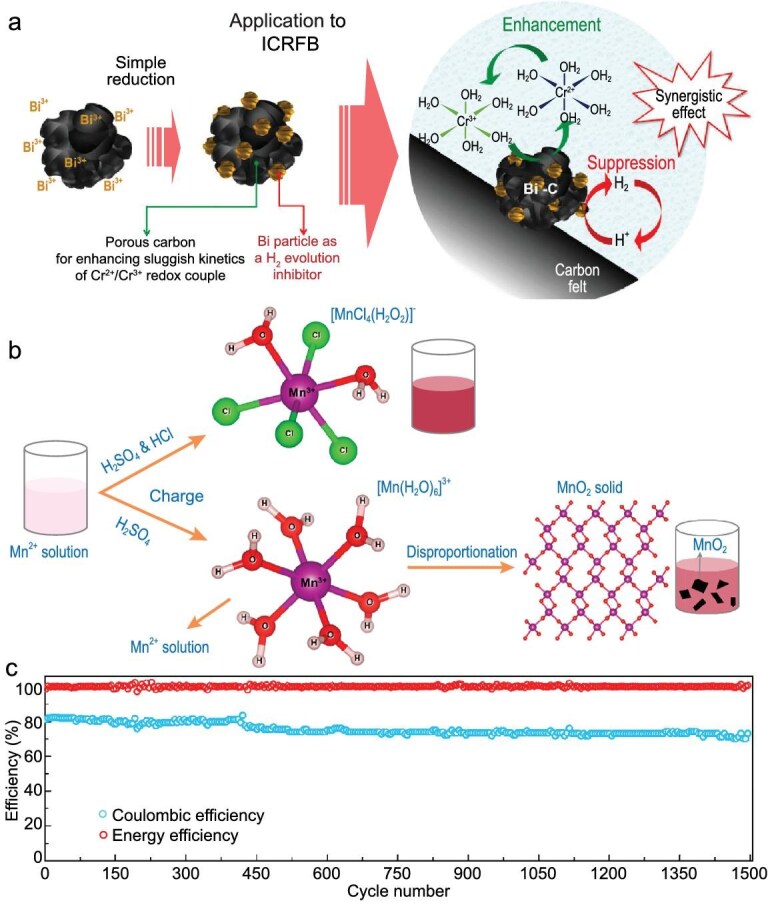
(a) Design concept of the bifunctional electrocatalysts in Fe-Cr ARFB system. Reproduced with permission from Ref. [83]. Copyright 2021 Elsevier B.V. (b) The molecular structures of [MnCl_4_(H_2_O)_2_]^−^ and [Mn(H_2_O)_6_]^3+^ complexes formed in Fe–Mn ARFB positive electrolyte. Reproduced with permission from Ref. [104]. Copyright 2022 American Chemical Society. (c) CE and EE of the Fe–S ARFB during a long cycling test. Reproduced with permission from Ref. [22]. Copyright 2021 Elsevier Inc.

Ahn *et al.* embedded bismuth nanoparticles into Ketjenblack carbon (KB) to synthesize a bifunctional catalyst that enhances Cr^2+^/Cr^3+^ redox kinetics while suppressing HER (Fig. 6a) [83]. The Bi/KB-coated negative electrode delivered an excellent electrochemical performance at 40 mA cm^−2^, with a CE of 98.2%, a VE of 88.2% and an EE of 86.5%. Xie *et al*. prepared a bifunctional bimetallic Pb/Bi catalyst, where bismuth effectively improved the reversibility of Cr^2+^/Cr^3+^ and lead with a high activation barrier inhibited HER [84].

Titanium nitride (TiN) has been used in ARFBs as catalysts due to its good catalytic activity and conductivity. Liu *et al.* reported that binder-free TiN nanorod arrays on 3D GF electrodes significantly improved cycling performance in Fe–Cr ARFB, achieving 93.0% CE, 90.4% VE and 84.1% EE at 80 mA cm^−2^ [85]. Additionally, titanium diboride catalyst deposited on carbon fibers further enhanced EE and battery cycling performance [86]. Carbon cloth (CC), valued for its high surface area and porosity, has been modified to address its limited active sites and poor reaction kinetics in flow batteries [87]. CC with uniform deposition low-cost indium catalyst particles used in the Fe–Cr ARFB platform can suppress HER and deliver a maximum power density of 356 mW cm^−2^ [88]. Polydopamine-assisted immobilization of the bismuth oxide catalyst-modified CC offers an excellent catalytic synergistic effect, which is attributed to increasing the active sites on the electrode surface and finally improve the kinetics of the Cr^3+^/Cr^2+^ redox reaction [89]. Silicic acid etching was used to create a dense nanoporous structure on CC, leading to a high average EE of 81.3% at 140 mA cm^−2^ in Fe–Cr ARFBs [90]. Similarly, silicon dioxide-modified GF with rich oxygen-containing functional groups and defect sites showed VE of 86.27% and EE of 79.66% with minimal capacity decay [91]. Moreover, benefiting from studying rayon- or polyacrylonitrile-modified CF, the electrochemical performance of Fe–Cr ARFBs had the potential to be further improved [78].

#### Electrolyte optimization

Another promising aspect to improving the performance of Fe–Cr ARFBs is to modify the electrolyte. Zhang *et al*. reported that an optimal electrolyte comprising 1.0 M FeCl_2_, 1.0 M CrCl_3_ and 3.0 M HCl achieved EE of 81.5% and 73.5% at 120 and 200 mA cm^−2^, respectively [92]. Cr^3+^ in acidic aqueous solution exists in three coordination structures: Cr(H_2_O)_6_^3+^, Cr(H_2_O)_5_Cl^2+^ and Cr(H_2_O)_4_Cl^2+^ [93]. The first has much lower electrochemical activity, leading to ion imbalance and capacity loss in Fe–Cr ARFBs [94]. Optimizing the electrolyte composition to increase the content of Cr(H_2_O)_5_Cl^2+^ and Cr(H_2_O)_4_Cl^2+^ can improve performance. Fe–Cr ARFBs using 1 M FeCl_2_ + 1.3 M CrCl_3_ + 3 M HCl electrolyte (E-1.3Cr) achieved a high EE of 84.51% at 80 mA cm^−2^ [95]. Another approach involves increasing chloride ion concentration in the electrolyte. Compared with NaCl and potassium chloride (KCl), lithium chloride was found to be the most effective additive, achieving an EE of 83.07% and a CE of 97.83% [96]. Chelating agents like diethylenetriaminepentaacetic acid have also improved solubility and stability of metal ions, achieving a remarkable CE of 100.0 ± 0.2% at 50 mA cm^–2^ at pH = 9 and a discharge power density of 216 mW cm^–2^ [97].

#### Membrane improvement

Nafion membrane is a cation-exchange membrane that has a wide range of applications in Fe–Cr ARFBs due to its high ionic conductivity and excellent chemical stability. However, Nafion membrane suffers from serious reactive ion cross-contamination, leading to decreased CE and capacity loss during the battery operation. Wang *et al.* prepared a series of sulfonated PBI membranes with precisely controlled sulfonation degree, which can effectively inhibit the crossover of Fe^3+^ and Cr^3+^, showing a capacity decay of only 1.11% per cycle [98]. So far, few studies on non-fluorinated ion exchange membrane materials in Fe–Cr ARFB systems have been reported. One type of covalent organic framework, sulfonated Schiff base network type (SSNW), was selected to modify sulfonated polyimide (SPI) to develop an SPI/SSNW composite separator with size exclusion effect, which delivers the decreased active ion permeability, exhibiting great application potential in Fe–Cr ARFBs [99]. Furthermore, sulfonic acid and iminium groups containing ionic covalent organic polymer membrane also facilitate the inhibition of ion penetration, demonstrating a capacity decay of 0.77% per cycle, which was significantly lower than that of recast Nafion (0.85%) [100]. The Grotthuss mechanism affects proton transport, and size exclusion and Donnan exclusion are used to elucidate the mechanism of ion selectivity in the membrane [3]. When designing the membrane for flow batteries, such as Fe–Cr ARFBs, which are plagued by the ligand-crossing issue, the focus should be on endowing the membranes with excellent ionic conductivity and ionic selectivity to construct flow batteries with high efficiency and low capacity decay.

#### Flow battery design

The conventional flow-through structure for earlier RFBs is detrimental to the uniform flow of electrolyte, which in turn leads to low EE. The advent of flow-field structure successfully combats this issue and exhibits a high EE of 79.6% at 65 °C in Fe–Cr ARFBs [101]. In the flow battery field, the application of 3D modelling can determine the optimal combination of channel and land dimensions, and machine learning (ML) can predict more efficient flow channel designs. Zhou and co-workers applied a 3D electrochemical flow-coupled model to determine the optimal flow channel spacing of 4 mm for the Fe–Cr ARFB system and developed an ML model with high prediction accuracy (R^2^ > 0.88) [102]. In addition, a multitasking ML model with higher prediction accuracy (R^2^ > 0.92) was developed to relate Fe–Cr ARFB characteristics to EE, CE and capacity [103].

### Other A-S half-iron ARFBs

Manganese is the 12th most abundant element, making it a cost-effective choice for ARFBs. Although the Fe^3+^/Fe^2+^//Mn^3+^/Mn^2+^ redox pair provides a net voltage of only 0.79 V (Equation 24), the high solubility of these metal precursors enhances energy density and electrolyte stability in Fe–Mn ARFBs. In 2020, Archana *et al.* demonstrated that MSA complexation stabilizes Mn^3+^ by increasing molecular volume and reducing molecular collisions. Under optimal conditions (4 M MSA, Nafion 117, 30 °C), the battery maintained over 95% CE and almost no capacity decay after 100 cycles at a current density of 7 mA cm^−2^ [20]. Wu *et al.* found that Cl^−^ complexation prevents manganese dioxide precipitation caused by manganese disproportionation, thereby supporting electrolyte reversibility (Fig. 6b) [104]. The assembled Fe–Mn battery achieved a high current density of 160 mA cm^−2^ with a projected cost of only $10.3 kWh^−1^, significantly lower than comparable Fe ARFB systems.


(24) \begin{eqnarray*}
&& \rm F{e}^{2 + } + \rm M{n}^{3 + } \rightleftharpoons \rm F{e}^{3 + } + \ \rm M{n}^{2 + }{\bf }\\
&&\quad{{\mathrm{E}}}^{\mathrm{0}}\ {\mathrm{ = 0}}{\mathrm{.79 \,V }}
\end{eqnarray*}

Similar efforts in Fe–V ARFBs have employed iron single atoms as redox catalysts loaded on graphene electrodes, achieving lower overpotential and higher EE [105]. Li *et al.* conducted extensive research on electrolyte optimization for aqueous Fe–V ARFBs, determining the general parameters for electrolyte fluids based on temperature stability [21]. Furthermore, membrane advancements, such as 15-μm thick HCl-doped PBI membranes, further improved discharge capacity and reduced operating costs of Fe–V ARFBs [106].

Sulfur-based systems have also gained traction due to their low cost and high theoretical capacity. Fe–S ARFBs offer excellent safety and flexibility. Long *et al.* developed a neutral Fe–S ARFB using polysulfides and high-concentration ferrocyanide as a redox pair, achieving 96.9% CE (Fig. 6c) after 1500 cycles at 20 mA cm⁻² and maintaining CE > 97% and EE > 80% in a 10-cell stack for 642 cycles (50 days) at 34 A [22]. Zou *et al.* developed an alkaline Fe–S ARFB with enhanced CE and stability. By leveraging the multi-ion effect, they increased [Fe(CN)_6_]^4−^ solubility up to 1.52 M, enabling stable cycling of an Fe–S ARFB with 1.3 M [Fe(CN)_6_]^4−^ for over 3152 h, achieving nearly 100% CE and a low capacity decay rate (0.11% per day) [107].

Organic redox couples have recently emerged as promising anolytes for iron-based ARFBs. Anthraquinone disulfonic acid (AQDS) has great potential for application in ARFBs due to its stability, solubility and redox potential. Yang *et al.* first reported an Fe–AQDS ARFB using ferric sulfate (a steel mill byproduct, $0.10/kg) and AQDS ($3/kg), demonstrating extraordinary longevity [23]. With their cost-effectiveness and charge-storage capabilities, Fe–AQDS ARFBs hold great potential in serving as a low-cost solution for large-scale energy storage.

## SUMMARY AND OUTLOOK

Currently, all-vanadium RFBs represent the most commercially advanced large-scale energy storage technology, with China having built the world's largest peaking power station at 175 MW/700 MWh. However, the instability in the supply and price of vanadium metal significantly limits the broader commercialization of all-vanadium systems. In contrast, iron-based flow batteries offer a more economically viable alternative, benefiting from the natural abundance, low cost and low toxicity of iron—features that make them particularly appealing for grid-scale deployment. Early attempts to commercialize iron-based systems, such as the Fe–Cr flow battery originally developed by Thaller, were explored by several companies during the 1980s and early 2000s. Currently, the only iron-based systems approaching commercialization are the all-iron (Fe–Fe) systems developed by companies such as ESS and VoltStorage. In addition, megawatt-scale demonstration platforms based on Fe – Zn ARFBs (in Canada and China) and Fe–V ARFBs (in Saudi Arabia) have been installed, highlighting the strong potential of these systems for future commercialization. Despite this progress, several technical challenges remain—namely, iron corrosion, zinc ion crossover and excessive hydrogen evolution. These issues significantly affect EE and cycle life and must be addressed to advance iron-based ARFBs from laboratory-scale research to industrial-scale deployment. Furthermore, reducing the cost of system installation and battery management infrastructure is essential for realizing the full cost advantages of iron-based ARFBs and supporting their path to commercialization.

Recent research efforts have focused on alternative iron-based chemistries to overcome these limitations. Table 3 summarizes key iron-based ARFBs discussed in this review and their performance characteristics. Advancements in iron-based redox couples, including Fe^2+^/Fe^3+^ and Fe^0^/Fe^2+^, are helping to improve electrochemical reversibility and efficiency. Efforts to integrate hybrid redox systems have further enhanced voltage stability and cycling performance. The use of high-surface-area porous carbon electrodes and electrocatalysts has significantly improved reaction kinetics and charge transfer efficiency. Innovative fabrication techniques, including 3D printing and carbon modifications, have further enhanced battery performance. Ligand-stabilized iron complexes and pH-tunable formulations have mitigated HER and metal precipitation issues, extending battery lifespan. Additionally, advanced electrolyte engineering and additives have played a crucial role in suppressing dendrite growth and improving ion solubility. The development of ion-selective, low-resistance membranes, such as AEMs and modified polymeric separators, has minimized ion crossover and enhanced chemical stability for long-term performance.

**Table 3.** Summary of the performance characteristics of typical iron-based ARFBs.

Types of iron-based ARFBs	Redox couples	Electrolyte	Electrode	Membrane	Battery type	Cycling life (h or cycles)	Current density (mA cm^-2^)	EE (%)	CE(%)	Capacity retention (%)	Capacity decay rate (%/cycle)	Ref
Acidic all-iron	Fe^2+^/Fe^0^ Fe^2+^/Fe^3+^	FeCl_2_–25 vol.% DMAc	CF + Fe sheet	Perfluorinated sulfonic acid membrane	Single cell	166 h	10	N/A	95	95.59	N/A	[42]
Acidic iron–zinc	Zn^2+^/Zn^0^ Fe^2+^/Fe^3+^	Fe–Py–TIRON	GF	Fumasep FAP-450	Single cell	325 h	40	60	94	N/A	N/A	[58]
Alkaline iron–zinc	Zn(OH)_4_^2^^−^/Zn^0^ [Fe(CN)_6_]^4^^−^/Fe(CN)_6_]^3^^−^	[Zn(OH) (EDTA) (OH_2_) _4_] ^3−^	CF	SPEEK	10-cell flow stack	950 h	40	82.35	97.86	N/A	N/A	[59]
Neutral iron–zinc	Zn^2+^/Zn^0^ Fe^2+^/Fe^3+^	Zn^2+^–Br^−^	CF	Nafion 212	3-cell flow stack	600 cycles	30	86.7	99.9	87.1	N/A	[64]
Acidic iron–tin	Sn^2+^/Sn^0^ Fe^2+^/Fe^3+^	0.5 M SnCl_2_ + 3.0 M HCl and 1.0 M FeCl_2_ + 3.0 M HCl	CF + carbon cloth	Nafion	Single cell	700 cycles	200	78	99	N/A	0.96	[13]
Neutral iron–tin	Sn^2+^/Sn^0^ [Fe(CN)_6_]^4^^−^/Fe(CN)_6_]^3^^−^	0.05 M Sn^2+^ + 3 M KCl and 0.2 M [Fe(CN)_6_]^3−^ + 3 M KCl	CF	Nafion 212	Single cell	120 cycles	10	N/A	97	60.8	0.51	[14]
Iron–lead	Pb^2+^/Pb^0^ Fe^2+^/Fe^3+^	1 M FeSO_4_ + 1.68 M H_2_SO_4_	GF	Porous PVDF-HFP membranes	Single cell	200 cycles	20	87.2	98.1	N/A	N/A	[77]
Iron–cadmium	Cd^2+^/Cd^0^ Fe^2+^/Fe^3+^	1.0 M FeCl_2_ + 0.5 M CdSO_4_ + 3.0 M HCl	GF	Nafion 212	Single cell	100 cycles	120	80.2	98.7	99.87	N/A	[16]
Iron–copper	Cu^2+^/Cu^0^ Fe^2+^/Fe^3+^	1.8 M CuCl_2_ + 0.01 M Bi_2_O_3_ + 2.4 M CaCl_2_ + 2.4 M HCl and 1.5 M FeCl_2_ + 0.01 M Bi_2_O_3_ + 3 M HCl	GF + carbon paper	Nafion 117	Single cell	10 cycles	20	35.24	89.18	N/A	N/A	[17]
Alkaline all-iron	Fe-NTMPA_2_/Fe-NTMPA_2_ [Fe(CN)_6_]^4^^−^/Fe(CN)_6_]^3^^−^	Fe- NTMPA_2_ and Fe–CN	Carbon paper	Nafion 212	Single cell	1000 cycles	20	87	100	96	0.0013	[80]
Iron–chromium	Cr^3+^/Cr^2+^ Fe^2+^/Fe^3+^	E-1.3Cr	CF	Nafion 212	Single cell	110 cycles	80	84.51	97.02	N/A	N/A	[95]
Iron–manganese	Mn^3+^/Mn^2+^ Fe^2+^/Fe^3+^	5 mL of 1 M MnSO_4_ + 3 M H_2_SO_4_ + 5 mL of 1 M FeCl_3_ + 3 M HCl	CF	SPEEK	Single cell	280 cycles	N/A	∼50	100	100	N/A	[104]
Iron–vanadium	V^3+^/V^2+^ Fe^2+^/Fe^3+^	1.5 M V^3+^ + 2 M HCl and 1.5 M Fe^2+^ + 2 M HCl	CF	*Meta*-PBI membrane	Single cell	10 cycles	80	∼80	N/A	N/A	0.3	[106]
Iron–sulfur	S_2_^2−^/S^2−^ [Fe(CN)_6_]^4^^−^/Fe(CN)_6_]^3^^−^	2.0 M K_2_S in 0.5 M KOH and 1.0 M K_4_[Fe(CN)_6_] + 1.0 M Na_4_[Fe(CN)_6_] in 0.5 M KOH	CF and sulfurized Ni foam electrode	K^+^-exchange N212	3-cell flow stack	3135 h	20	N/A	100	N/A	0.0166	[107]
Iron–AQDS	FeSO_4_/Fe_2_(SO_4_)_3_ AQDS/AQDSH_2_	0.67 M FeSO_4_ + 0.33 M AQDS + 2 M H_2_SO_4_	Multi-wall carbon nanotube-modified GF	Nafion 212	Single cell	500 cycles	200	N/A	99.63	N/A	0.000 076	[23]

Despite these advancements, iron-based ARFBs still face major challenges that hinder their commercial viability. In acidic all-iron ARFBs and other deposition-based systems, HER and dendrite formation remain critical barriers to achieving long-term cycling stability and high CE. These issues lead to capacity loss, increased cell resistance and potential safety concerns. In A-S iron ARFBs, managing crossover effects and maintaining electrolyte stability over extended cycles is a challenge. Ligand degradation, ion crossover and electrolyte viscosity changes affect overall battery efficiency and reliability.

While laboratory-scale studies have demonstrated promising performance, most research is still conducted on single-flow cells under controlled conditions. Translating these advancements into pilot-scale systems introduces additional complexities, including optimizing flow stack design, flow rate control and large-scale electrolyte management. Factors such as electrode architecture, flow field distribution, and electrolyte replenishment significantly influence real-world battery performance and scalability.

To bridge the gap between laboratory-scale research and practical deployment, future studies should focus on the following key areas.

### Materials innovation and mechanistic understanding

Advancements in materials science are crucial for addressing challenges such as electrolyte stability, electrode kinetics and membrane selectivity. Research should focus on developing novel ligands and additives to enhance Fe-ion solubility, suppress HER and prevent dendrite formation. For all-iron flow batteries, electrolyte engineering is particularly important to mitigate HER, which competes with iron redox reactions. Additionally, optimizing carbon-based electrodes through surface modifications or catalyst coatings can enhance charge transfer efficiency. Membrane improvements, including AEMs, proton exchange membranes and intrinsic microporous polymers, aim to reduce ion crossover and extend cycle life [108]. Understanding ion transport mechanisms and selectivity behaviour within these membranes is crucial for optimizing performance over extended cycles.

### Flow battery system design and manufacturing

Flow system design significantly influences mass transport, pressure distribution and overall electrochemical efficiency. Optimizing parameters such as flow field patterns (e.g. serpentine, interdigitated, parallel), flow rate and temperature control is critical for stable operation. Efficient flow distribution reduces polarization losses and enhances reactant utilization. Additional design improvements, such as RC compartments and catalyst-loaded separators, can mitigate side reactions like HER, improving Coulombic efficiency. The use of 3D-printed flow frames enables precise customization of flow fields, reducing manufacturing costs [109]. ML models can dynamically optimize flow dynamics and operational parameters to maximize system performance and longevity [110].

### New electrochemistry explorations

Beyond conventional Fe²⁺/Fe³⁺ redox chemistry, hybrid redox couples could improve energy density and cycling stability. Integrating iron species with vanadium, organic molecules or halogens could expand the electrochemical operating window. Decoupled hybrid architectures separating charge storage from electrochemical conversion may enhance efficiency and operational flexibility. Further studies on alternative iron redox species and complexation strategies could unlock new electrolyte formulations with improved solubility and electrochemical stability.

### Interdisciplinary strategies

Advanced characterization techniques, such as synchrotron X-ray tomography [111], Raman spectroscopy, Fourier transform infrared spectroscopy and X-ray fluorescence microscopy [112], provide real-time insights into HER, dendrite formation and redox kinetics. Artificial intelligence (AI)-driven approaches can accelerate material discovery by predicting optimal electrolyte additives, electrode materials and membrane compositions. High-throughput electrochemical testing platforms can rapidly evaluate material combinations, expediting the development of next-generation iron-based ARFBs.

Iron-based ARFBs hold immense potential as a cost-effective and scalable energy storage solution, but significant advancements are still required to achieve commercial competitiveness with vanadium-based systems. Addressing fundamental challenges related to electrolyte stability, dendrite formation, ion crossover and large-scale system integration will be critical for their future development. Despite these challenges, iron-based ARFBs have strong potential for low-cost long-duration energy storage applications. By leveraging interdisciplinary approaches—including materials science, electrochemistry, fluid dynamics, advanced characterization techniques and AI-driven optimization—researchers can accelerate the path toward practical and commercially viable iron-based ARFBs. Overcoming these barriers will unlock a sustainable, high-performance and low-cost energy storage technology capable of supporting the global transition to renewable energy. Future commercialization efforts will require advancements in materials optimization, system design and pilot-scale validation to bridge the gap between laboratory research and real-world deployment. Increased collaboration between academia, industry and government initiatives could accelerate the path toward market adoption.
